# Looking for a Taste of Home: A Qualitative Study of the Health Implications of the Diets of Australian - Based Southeast Asian Students

**DOI:** 10.5539/gjhs.v8n3p101

**Published:** 2015-07-13

**Authors:** Jodie H. Leu, Cathy Banwell

**Affiliations:** 1 ANU Medical School, The Australian National University, Canberra, Australian Capital Territory, Australia; 2 National Centre for Epidemiology and Population Health, The Australian National University, Canberra, Australian Capital Territory, Australia

**Keywords:** Southeast Asia, ethnography, health and well-being, health promotion, semi-structured interviews, qualitative

## Abstract

**Purpose:**

To investigate potential dietary changes among Southeast Asian international students living in self-catered accommodation while studying abroad and to consider implications for their health.

**Design:**

Participants were interviewed about their food preferences and behaviours in their home countries and during their undergraduate studies at the Australian National University.

**Setting:**

A university in Australia

**Participants:**

Study participants were full-time undergraduate students over 18 years of age from Southeast Asian countries studying at the Australian National University for at least one year, and living at self-catered accommodation.

**Methods:**

Thirty-one, in-depth, face-to-face qualitative interviews concerning usual diets were collected over a three month period in 2013. Interviews were coded and analysed with the aid of a computer program Atlas.ti.

**Results:**

The macro-nutrient content of Southeast Asian international students’ diets did not change a great deal when they moved to Australia. Most students replaced some preferred foods on occasions because they either could not afford them, they were not available or they lacked time to prepare them. These dietary changes were not necessarily reflected in changes to students’ weights and most students considered that they were as healthy as when they lived at home.

**Conclusion:**

As students’ adapt to a new food environment they reflexively manage potential health risks. Strong student networks and an accessible and healthy food environment would support students to make healthy dietary choices although additional information about healthy diets could facilitate this further.

## 1. Introduction

Each year an increasing number of students leave home to pursue higher education. In 2011, approximately 3.5 million students mainly from Asian countries such as China, India and Korea (Altbach et al., 2009; Coughlan, 2011) went abroad to study. In Australia, international students make up to 22% of the university population (Australian Government Department of Education and Training, 2014). These students face challenges due to their youth and inexperience in self-care. They are required to rapidly learn how to juggle their studies, cope with acculturation, form new friendship groups, and adapt to a new lifestyle. While challenging, these experiences encourage students to develop an adult and independent identity. Meanwhile, the habits and preferences formed during this period may have a lasting influence on their diets and health (Steptoe et al., 2002; Cluskey & Grobe, 2009; Ansari et al., 2012; Fyler et al., 2014).

Studying overseas has been implicated in increased health risks. Students from countries such as China and India reduced their vegetable and meat intake when studying in the US and UK (Pan et al., 1999; Reeves & Henry, 2000; Papadaki & Scott, 2002; Satia-Abouta et al., 2002; Danquah et al., 2009; Almohanna, 2010; Trapp et al., 2011). Additionally, students are known to often turn to junk and processed foods (Racette et al., 2008; Strong et al., 2008). These dietary changes are similar to a shift to the modern western diet that is energy-dense, high in fat, sugars and low in vitamins, minerals and other valuable micronutrients (World Health Organisation, 2013). It is associated with overweight and obesity, poor physical and mental health and other non-communicable diseases (Hill & Weaver, 1991; Stoppani, 2007; Challem, 2008; Luntz, 2009; Smith, 2011). Furthermore, as students spend much time studying, they tend to have sedentary lifestyles that may exacerbate weight gain or result in poorer health (Schmidt, 2012).

Many factors have been identified that influence the type of food students are familiar with and which subsequently guide their tastes when they study abroad. These factors include their home food culture and domestic diets which are usually developed over childhood (Messer, 1984; Kittler & Sucher, 2008; National Consumers League, 2009; Amos & Lordly, 2014) and are deeply associated with identity making (Mead, 2007). In a new environment, factors such as affordability, accessibility and palatability of foods may also influence their dietary practices. For example, students are likely to have limited finances and they may find that preferred and familiar foods are either not available or too expensive (White & Kokotsaki, 2004; Certeau, 2007). Finally, as food preparation is often time consuming, students may resort to eating fast foods, instant meals or eat out particularly when under time pressures such as during exam and assessment periods. Ultimately all these factors can contribute to dietary changes with potential health impacts.

Until now, international student populations have been largely neglected in research into the health of young adults despite the growing numbers of students worldwide (Altbach et al., 2009; Coughlan, 2011). Most existing studies have been conducted in the US and UK and focus on Chinese and Indian students while other student nationalities are often overlooked. However, at Australian universities Southeast Asian students are an increasingly large and important group, making up the majority of remaining international student nationalities (The Australian National University, 2011). This paper investigates the significance and consequences of, and these students’ responses to, factors that may influence their diets in an Australian university environment. Our aim is to develop an understanding of these students’ perspectives on dietary changes to identify and emphasize areas to assist them in managing their lives and health in a new environment.

## 2. Method

### 2.1 Participant Characteristics and Sampling Procedures

**Figure 1:**
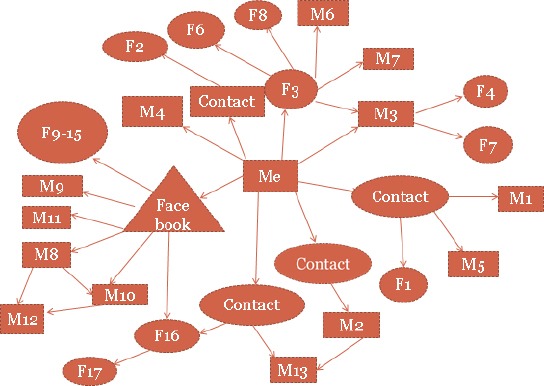
Diagram showing how participants were recruited through convenience and snowball sampling

Research participants were full-time undergraduate students from Southeast Asian countries aged 18 and over studying at the Australian National University for at least one year, and residing in self-catered accommodation. Initially, participants were purposely selected via the author's (JL’s) access to several Southeast Asian student organisations and at events throughout the campus. Subsequent participants were recruited by snowball sampling. In addition, Facebook was used as a media platform where details of the study were posted onto two Facebook groups (as shown in Figure 1). Singaporean and Malaysian Chinese students were among the first to participate in the study followed by students from other Southeast Asian countries.

### 2.2 Data Collection and Analysis

**Figure 2:**
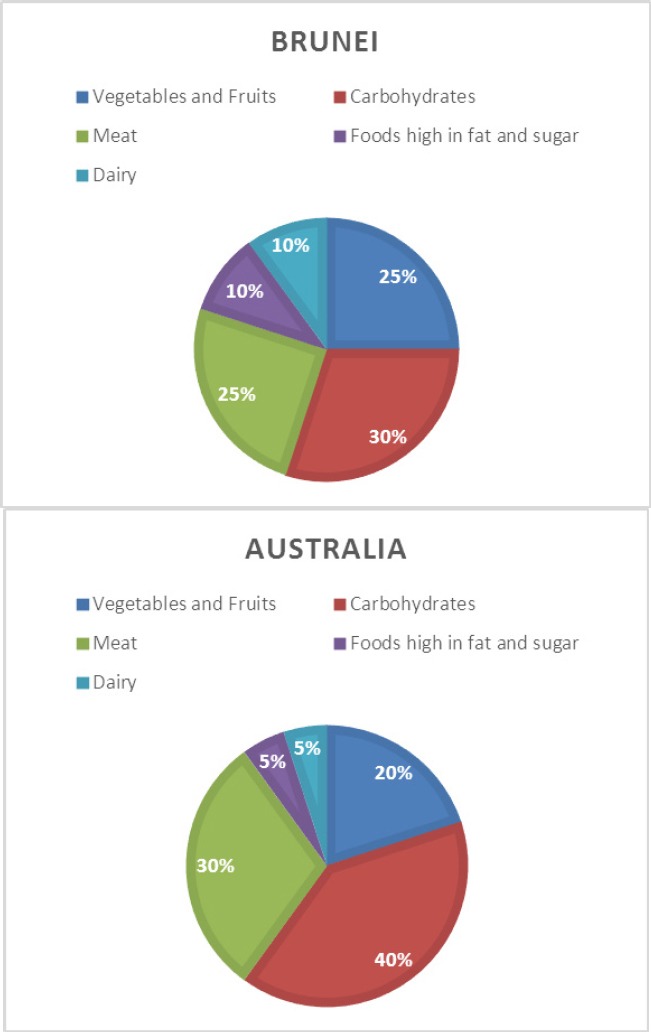
An example of a student's perception of the macro-composition of their dietary changes

This study aimed to investigate the question: ‘How do the diets of the international student population change after commencing study at the Australian National University?’ with a specific focus on issues of affordability, accessibility, palatability, time constaints and weight and health implications. A semi-structured interview guide was developed based on relevant work by Eckermann (1997) and Hubert (2004). Open-ended questions were used to explore students’ understandings and perceptions of their food preferences and behaviours both in their home countries and in Australia; their awareness of health campaigns in both settings; and their current exercise patterns and satisfaction with their weight. In addition, basic socio-demographic and health-related information such as perceived weight change, and self-rated health status was also collected (Miilunpalo et al., 1997; Mikolajczyk et al., 2008). Changes in macro nutrient intake were collected by asking students to illustrate the macro-composition of their old and new diets on pie charts (see Figure 2 for an example).

The lead author (JL) conducted thirty-one interviews in English which were collected over a three month period in 2013 at a time and place convenient to the participant. Interviewing stopped once saturation of the main topic was achieved meaning that very little new information was gained in later interviews (Ulin et al., 2005; Denscombe, 2010). Interviews were professionally transcribed and uploaded to Atlas.ti, a computer program that facilitates the coding process. The lead author read and re-read the transcripts to identify key concepts and codes related to food preferences, food practices, and dietary changes (Ulin, Robinson et al., 2005) and discussed the coding framework with the second author. Emergent themes were checked against existing literature and new themes were identified using a modified grounded theory approach (Pope & Mays, 2006).

### 2.3 Ethics and Informed Consent

Ethics clearance for the study was gained from ANU Human Research Ethics Committee (ANU HREC). Before participating in the interview, participants were fully informed about the study, their right to refuse to answer any questions and to withdraw from the study at any time. Each participant was also given a pseudonym to maintain confidentiality.

## 3. Results

### 3.1 Sample

The participants included more female than male students with most identifying Singapore as their country of origin followed by Malaysia and Vietnam. This mirrors the composition of international students at the university with 35.38% of international students coming from Southeast Asia (The Australian National University, 2011).

**Table 1:** Participants’ Country of origin

Country of Origin	Participants	

	Male	Female
**Indonesia**	2	0
**Malaysia**	1	4
**Singapore**	7	7
**Thailand**	1	1
**Vietnam**	0	4
**Other SE Asian countries**	2	2
**Total**	13	18

NB: Participants were studying 19 different degrees with the largest number (9) doing a Bachelor of Commerce.

### 3.2 Affordability

Despite similarities in geographical location, each Southeast Asian country has its own culinary culture (Yen-Ho, 1995; Tan, 2011). While home culinary cultures formed an important part of students’ food choices, this paper concentrates on issues that are relevant to most Southeast Asian students.

In this study, most participants had not lived away from home previously and had to learn for the first time how to manage their everyday living costs. Their budgets ranged from $20-200 per week. Their average expenditure on food per week was AUD81.54 for males and AUD85.42 for females. Students said they experienced ‘price shock’ because Australian food prices were several times higher than those they were used to or had expected.

Oh my gosh, like, why do I need to spend this much for… daily? So like… So I don’t like to cook… [I] start cooking every day which I never did when I was in Malaysia. I never cooked at all when I was in Malaysia so I started cooking. And I didn’t do that immediately so it was like a forced change that I had to do, like immediately. And then I guess like buying stuff I guess was like, oh my god. But just prices in general. - (Nila)

However, they developed strategies to manage. Students took time to compare prices between the major supermarket chains in Canberra. Mia explained that her and her friends tried to save money when buying food and said *“we actually take pictures of the products to see…”* Regardless of their socioeconomic backgrounds most students’ searched for cheap and fresh items when shopping for food. Nevertheless, some participants would weigh up the benefits of paying a little more for items that taste better or have more nutritional value.

Um, I usually pick the cheapest one but it depends on the quality as well. Like, if it's cheap but very bad quality, I don’t use it. Sometime the beef is on a special price, like $5 a kilogram but it's already black. I just don’t use that. - (Will)

Imported ethnic or cultural foods tend to be sold in lower quantities at higher prices (White & Kokotsaki, 2004). Thus, students often reserved ‘cultural foods’ for special occasions such as potlucks and group gatherings due to the cost and seasonality of ingredients and the amount of time needed to make the foods. Participants’ also switched to other foods when the item they preferred was too expensive for their budget. For instance, Kaylee had come from a seaside town and was used to eating seafood daily but in Canberra she switched to meat as a source of protein instead. There were also participants who bought frozen vegetables and meats as these items could be stored for longer and cost less; though this was affected by accessible fridge space at their accommodation.

Many reasonably priced Asian restaurants are located close to the Australian National University to cater for the large number of Asian students. The frequency of eating out at restaurants varied greatly between participants and depended on factors such as the occasion, and whether they had additional money to spend. Most participants went to restaurants for birthdays or large gatherings of friends where the main course cost between AUD$15-30. In general, these shared meals, while more expensive than eating at their accommodation, were important as they allowed participants’ to bond with others over food, thereby creating their own support systems that not only aided the process of acculturation but helped form friendships during their studies in Canberra (Amos & Lordly, 2014). Tran said it was also considered impolite to refuse invitations to eat out with friends due to the lack of funds.

I don’t usually eat outside, I just buy food like once or twice a week, but when friends tried to gather round, it wouldn’t be nice to say, “Oh I’m not gonna go out it's too expensive” so I just go - (Tran)

Students also had an opportunity to eat interesting food if they obtained part-time employment at restaurants to supplement their incomes or contribute to the cost of their studies.

I used to work at Thai restaurants so I get free Thai food all the time, really spicy food - (Jenny)

Regardless of their socioeconomic backgrounds, students tended to shop economically but were reluctant to sacrifice the quality of goods while doing so. However, it was unclear how much students relied on cheap foods such as instant noodles and other junk foods, as this depended on their moods and personal preferences which fluctuated and at times correlated with time scarcity and intensity of studies.

### 3.3 Accessibility and Availability

As globalization and migration patterns develop, Southeast Asian food cultures have been growing and evolving (Yen-Ho, 1995; Tan, 2011). Southeast Asian restaurants are common in Australia to cater to numerous Southeast Asian immigrants wanting to make and eat their own cultural foods as a connection with their home country (Duruz, 2007; Amos & Lordly, 2014). This growth has also promoted a trend in fusion foods (Tan, 2011), although this was not always viewed positively by participants.

When I miss Thai food like in my hometown, I can’t really find like a good Thai food around… they are more Western Thai? You know what I mean? – (Will)

That just looks like some kind of bastardized fusion of different things and they’re not really right. – (Brendan)

In the past, eating foreign foods in Australia was a luxury since most of the ingredients were imported thereby making these foods more expensive (White & Kokotsaki, 2004). However, foreign ingredients have become more accessible and somewhat more affordable recently due to higher demands. For instance, there are now aisles in mainstream Australian supermarkets devoted to condiments, ingredients, and prepacked or instant meals from overseas. Along with an influx of Asian grocery stores, this has allowed students to find ingredients to make their own cultural foods. However, a young woman suggested that locally bought foreign ingredients does not always taste authentic and can lead students to be disappointed in dishes they have prepared themselves.

Throwing away food is fine for me but food that you cook it's like “oh shit”. I’ve been spending an hour or two doing that and now I’m throwing it away. It's kind of sad but you know. – (Tan)

Nevertheless, many students have adapted their diets to Australian conditions. They bought more salads, cereals and ingredients to make western foods that they enjoy as they are more easily available and easily prepared. Others however, still preferred to get some items from Asian grocery stores and these choices reflect how strongly participants adhere to their cultural identity and cultural foods (Anderson, 2005; Amos & Lordly, 2014).

I try to look for Halal, like in [supermarket] where we can get Halal chicken. But I’m not really a fan of chicken…‘cause I really like beef, and the only one you can get beef from is in I think [a far suburb]…I went though, with [Friend] once, and it's usually expensive, probably ‘cause it's the only place who sells it, and that was the only time I went… – (Putri)

Canberra is renowned as a car-dependent city but most students cannot afford their own cars. It has a comparatively poor public bus service (to other major Australian cities) which students are forced to use to get to grocery stores in the city centre or elsewhere. Some students also walk about 20 minutes’ from the University to the city centre whilst others ride their bikes.

I ride a lot, and my bike is my baby, seriously… with a bike, the trick is to fill up the basket [with food] and hang the rest of the stuff on the handles and then push it because if you ride it you’ll definitely fall, and that way you can actually put more stuff and do more work – (Jenny)

As participants did not always have large amounts of free time, some of the cheaper markets and some speciality items outside the city centre were inaccessible for those who had no convenient and reliable transport. Nonetheless, participants’ were willing to take the time to travel the extra distance by bus or car to seek out a particular ingredient. Women did not like to shop alone at night for safety reasons despite the short distance from the student accommodations to the city. They preferred to be accompanied by other friends or to shop during the day. Nevertheless, despite these limitations, most managed to access culturally valued foods.

### 3.4 Palatability

Many participants remarked that their home dietary culture strongly influenced their adult dietary preferences and behaviours. They preferred to eat the kinds of food they ate at home. For instance, many participants ate rice with most meals as it has been a part of their diets since they were very young. However, they also tried to be receptive to new foods which they could incorporate into their dietary repertoire (Anderson, 2005). Although taste or palatability has been identified as an important factor influencing the purchasing and making of foods, (Franciscy et al., 2004), this was not found to be the case in this study to the same extent. Students considered it a luxury to have the time and money to make or buy good tasting foods. Participants said they were sometimes in ‘survival mode’ when they ate food for sustenance and to relieve hunger rather than worrying about its taste. Consequently, the taste of food was important but less so that than other factors.

### 3.5 Time Scarcity

It takes time and practice for inexperienced students to learn to cook for themselves. Tan, a second year student, explained how she learnt:

Okay first year I can’t cook at all. Like first time I came here I don’t really know how to use a knife. I don’t really know how to use knives and people are like, ‘Oh no I cook for you…’ My friend has been cooking for me for a while since first year… So first semester, people cook for me and then I tried to learn, they tried to teach me and like in that period of time and then second year I improved and now I can cook. - (Tan)

Participants, like Tan, with no prior cooking experiences struggled at first while others had been taught the basics like boiling water, cutting techniques and some simple recipes by their parents or housekeepers before commencing their studies. Often, participants would call their mothers or friends to ask how to cook certain foods or consult the internet. Both male and female students became better cooks over time. However, this did not always mean that they cooked healthy meals as time was also a crucial factor. Most students made simple stir-fries, pan-fried meats, soups, rice and noodles with various types of seasoning with the time taken ranging from 20 minutes to one hour, including eating and cleaning.

At first, most participants’ juggled their daily living requirements with university demands whilst coping with homesickness. Over time, many developed strategies to improve their time management use. These included using familiar easy recipes, eating out if they were pressed for time particularly during exams and assessment times, cooking in bulk and storing food, and sharing cooking with friends. While cooking with friends was usually seen as a social activity (Amos & Lordly, 2014), some participants’ also did this to provide more nutritionally balanced meals through the inclusion of a greater range of dishes and reduced costs (Ball & Brown, 2012); around AUD5 per person per meal was considered reasonable. However, this required complex scheduling to include a number of people as well as the need to accommodate the varying tastes and dietary requirements. Hence, some participants found that it was easier to cook by themselves. Overall, most participants’ found cooking to be therapeutic and they enjoyed planning and cooking their meals, provided it did not take too long.

### 3.6 Health

According to an analysis of their pie chart diagrams, the macro-nutrient content of participants’ diets did not appear to change greatly. Only 8 (26%) of participants indicated that they ate fewer vegetables and fruits and increased their meat and carbohydrate intake. Other participants (11; 35.5%) said they made minimal or no changes in their diets and others thought that their diets were healthier (12, 38.7%), meaning that they had increased their fruit and vegetable intake and consumed less meat and carbohydrates. Women's diets were usually healthier while males tended to have similar or less healthy diets compared to their home diets. Most participants still thought that they could improve their diets by eating more fruits and vegetables although they also acknowledged that this would be more costly.

Healthier foods are actually more expensive…Natural foods are actually more expensive – Leny

Because processed foods are cheaper than fresh foods, we expected that students who spent less on food would have less healthy diets and lower self-rated health. However, (Table 2) men who had lower self-rated health spent more on food than those with higher self-rated health. In contrast, women who rated their health poorly spent less. Overall, most participants’ assessed their self-rated health positively (Table 2). In a small, cross-sectional study such as this it is difficult to draw any conclusions although this finding suggests that men and women responded to budgetary constraints differently.

**Table 2:** Participants’ Self-rated health and average food expenditure per week

Self-Rated Health	Poor and Fair (N=9 29%)	Good (N = 14 45.2%)	Very Good & Excellent (N= 8 25.8%)
**Males**	5	3	5
**Females**	4	11	3
**Males’ Average Food Expenditure/Week (AUD)**	97.00	78.33	72.50
**Females’ Average Food Expenditure/Week (AUD)**	53.34	65.68	142.5

In comparison to European counterparts, fewer participants’ felt that their health was very good (12.9% vs. 35.8%) and more felt that their health was only fair (25.8% vs. 8.9%) (Miilunpalo, Vuori, Oja, Pasanen, & Urponen, 1997). In the interviews participants who rated their health highly attributed this to frequent exercise rather than diet. Those who thought that their diets were healthier did not necessarily have better self-rated health.

Male participants’ BMIs ranged between 20.5 to 28.4 and females ranged between 17.8 to 28.9 (BMI = Weight (kg)/Height^2^ (m)). Male and female students within a healthy BMI range generally spent comparatively modestly on food. However, a heavier BMI category was linked with greater expenditure on food among men and a lower expenditure by women suggesting once again gendered behaviours around food purchasing.

**Table 3:** Participants’ BMI and average food expenditure per week

BMI Classification	Underweight <18.5 (N = 2, 6.5%)	Healthy Weight 18.5 - 23.9 (N = 20, 64.5%)	Overweight 24-26.9 (N = 5, 16.1%)	Obese >27 (N = 4, 12.9%)
**Males**	0	7	4	2
**Females**	2	13	1	2
**Males’ Average Food Expenditure/Week**	n/a	72.86	85.00	105.00
**Females’ Average Food Expenditure/Week**	92.5	78.27	75.00	55.00

NB: The ranges are based on Asian BMI cut-offs (Kanazawa, Yoshiike et al. 2002, Joslin Diabetes Center 2010).

## 4. Discussion

This study illustrates the way in which Southeast Asian students at the Australian National University have adapted to their new food environment. Despite initial difficulties, they have learned to manage issues of food affordability, accessibility and palatability and over time develop a reasonably healthy diet. A proportion of them thought that their diet was healthier than it had been in their home countries. Most existing studies find that international student diets are less healthy than their home diets (Pan et al., 1999; Reeves & Henry, 2000; Papadaki & Scott, 2002; Satia-Abouta et al., 2002; Danquah et al., 2009; Almohanna, 2010; Ansari et al., 2012).

Existing literature shows that many international students (not in Australia) have unhealthy dietary practices including reduced intakes of vegetables, decreased or increased meat consumption in relation to affordability, and eating less due to budgetary constraints. They also skip breakfast due to class timetables meaning they eat only two meals a day. This is often associated with cost as the over-riding factor in influencing food preferences and choices along with availability and palatability (Pan et al., 1999; Reeves & Henry, 2000; Papadaki & Scott, 2002; Certeau, 2007; Danquah et al., 2009; Almohanna, 2010; Peltzer & Pengpid, 2015). Additionally, securing food has been identified as one of the factors that students worry about along with finances and loneliness (Gu et al., 2010). Other factors such as home cultures and time scarcity are also influential. Our participants were able to manage issues of affordability and availability and still purchase the kinds foods that they wanted to eat or make. These food choices were guided by familiar flavours from their home countries; though many were also willing to try and integrate other cuisines that they are not familiar with into their diets.

While palatability is thought to be a large factor affecting diets (Franciscy et al., 2004; Anderson, 2005), it was not always a determining factor for these students as there were times when food was thought to be only necessary for survival. The palatability of foods was sometimes less important than the time available to cook and eat. Students tended to eat premade meals and fast food when pressed for time during exam periods.

Our participants mainly rated themselves healthy although half the males were overweight or obese. Overall, their expenditure on food per week demonstrated that maintaining good self-rated health did not necessarily entail a high budget. Most participants felt that they could achieve an adequate diet on their budgets.

Weekly food expenditures pointed to differences in how men and women managed their diets within a tight budget. Women who were overweight or obese spent less on food than women of a normal BMI. Men who were overweight or obese spent considerably more than men with a normal BMI. This could be due to a variety of reasons such as: men eating much larger quantities to satisfy their hunger, buying more premade foods and being more likely to eat out than females (National Consumers League, 2009; de Saint Pol, 2014) while women on a low budget may be eating cheaper, less healthy and more energy dense foods or snacking more on cheap junk foods. It is possible that women who were concerned about their weight were spending less on food by buying smaller quantities. Women in the study with high self-rated health spent more on food than other women. Men with high self-rated health spent less than other men. Research (de Saint Pol, 2014), suggests that women may have more time or delegate more time to cook or shop often for healthier food options. In addition, men are more likely to over-estimate their health status than women (Wardle et al., 2006). These differences could also be due to personal physiological traits such as metabolism rates and family health history (Magkos & Mittendorfer, 2009). Additionally, while men and women with higher educational backgrounds tend to be more self-conscious and knowledgeable about their diets, differences in expenditure could also be due to the fact that women are more likely to be affected by sociocultural needs to be slimmer. Personal economic resources may also be a factor in food expenditure differences and health outcomes as those who have more money to spend are more likely to have access to better quality products (Cheung et al., 2011; Joh et al., 2013).

Based on data collected through their pie charts, it appears that participants mostly managed to maintain or even improve their diets by consuming more fruit and vegetables and fruit and minimizing carbohydrates, compared to when they were living in their home countries. However, this does not imply that their home diets were unhealthy. Indeed, traditional Southeast Asian diets are generally considered healthy and it is has only been since the widespread adoption of a more westernised and energy dense diet in these countries that there has been concern about diet-related health (Bengoa, 2001; Popkin & Gordon-Larsen, 2004; Popkin, 2004; Satia, 2010). Additionally, some participants also mentioned that obesity prevalence was not an issue in their home countries. For instance, in Vietnam, the prevalence of obesity was 1.6% for the population aged 20 and above (World Health Organisation, 2011).

Due to time and resource limitations in this study we were unable to measure caloric intakes, procure detailed food diaries, or measure food quantities and types accurately to obtain a clearer analysis on their cross-country dietary changes. Our findings related to food budgets, weight and health suggest that further qualitative and quantitative research would be useful. As this was a small qualitative study, the findings are not generalizable to the student population as a whole. It does provide however, an in-depth understanding of the factors related to dietary changes and health related outcomes that occur when Southeast Asian students move to Australia.

While there is an abundance of information on healthy diets and behaviours available through Australian media, there are no active campaigns or support for international students. However some students in this study sought information because of their desire to be healthy suggesting that provision of diet and exercise information targeting overseas student groups at universities could be helpful.

## 5. Conclusion

Overall, this research found that most Southeast Asian students considered that their diets in Australia were as healthy as or healthier than in their home countries in terms of macro nutrients and food groups consumed. Due to issues of affordability, availability of cooking materials, and lack of food preparation time, most students found alternatives for some home foods. These Southeast Asian international students seemed to have better diets than those reported for other international student populations. BMI and self-rated health did not worsen due to dietary changes as suggested in previous literature. This may be because our student participants lived in self-catered accommodation, and could control their diets, cooking methods, and lifestyle. This dietary control was supported by relatively easy access to imported Asian foods and a range of fruit and vegetables available in nearby local supermarkets and shops. Another contributing factor to students’ physical and mental well-being was the relationships they formed with other students with whom they cooked, shared chores and meals, and support. While catered accommodation frees time for students to study it may not provide as healthy an environment due to limited food choices and repetitive menus (Pliner & Saunders, 2008; Cluskey & Grobe, 2009). This perception was supported by some participants who had previously lived in catered accommodation. This study has implications not only for students’ health but also for their ability to study and make the most of their opportunities while engaging in studies overseas.

As the factors of affordability, accessibility, availability, time scarcity, and palatability have been identified as potential barriers to achieving healthier diets, future studies could be done on how university facilities and related food provision services can help students achieve better diets and health in general. For instance, experimenting with trial cooking workshops, free exercise classes and information on how to prepare healthy meals targeted at the student population could help in finding solutions to promote better health and well-being. Without any interventions, long term unhealthy diets could affect students later on in life due to the association between unhealthy diets and the onset of non-communicable diseases.
